# IMPACT OF SUBSIDY ON THE USE OF PERSONALIZED MEDICINE IN BREAST CANCER

**DOI:** 10.1093/geroni/igac059.851

**Published:** 2022-12-20

**Authors:** Cynthia Chen, Jue Tao Lim, Swee Ho Lim, Ern Yu Tan, Veronique Kiak Mien Tan, Su Ming Tan, Tira Tan, Mikael Hartman

**Affiliations:** National University of Singapore, Singapore, Singapore; National University of Singapore, Singapore, Singapore; KK Women's and Children's Hospital, Singapore, Singapore; Tan Tock Seng Hospital, Singapore, Singapore; Singapore General Hospital, Singapore, Singapore; Changi General Hospital, Singapore, Singapore; National Cancer Centre Singapore, Singapore, Singapore; NUS Yong Loo Lin School of Medicine, Singapore, Singapore

## Abstract

Advances in adjuvant therapy have led to increased survival rates after cancer prognosis. Herceptin, a targeted therapy, had first been introduced to Singapore in 2006. We aimed to assess whether subsidies for Herceptin from 2012 will lead to changes in uptake among Human Epidermal Growth Factor Receptor 2 (HER2) positive patients by socio-economic groups. Two-level random-intercept logistic regression was used to model diagnostic test and Herceptin uptake using the Singapore Breast Cancer Cohort from 2006 to 2018, adjusting for covariates such as education, housing type and marital status before and after subsidies. Interrupted time series (ITS) analysis was used to evaluate the impact of Herceptin subsidy on treatment uptake. The concentration index was also computed to measure inequality in uptake by ethnicity and education. We found that the odds of diagnostic testing were not associated with socioeconomic factors. However, before subsidies, the highest education attained (OR = 4.57, 95% CI= (1.90, 11.02), P< 0.01) significantly increased the odds of Herceptin uptake. These odds were levelled after the introduction of subsidies to Herceptin treatment in 2012. After subsidy, we also found that Herceptin uptake increased significantly by 11.4% (95% CI= (3.47%, 19.4%), P=0.016). Also, inequality of Herceptin use decreased especially amongst the Indians, where at least 40% were used in the higher educated group prior to the subsidy. Subsidies have lowered the barriers to Herceptin uptake for marginalized individuals. Having targeted subsidies for socio-economically disadvantaged groups may work more efficiently in providing ease of access than a blanket subsidy in Herceptin.

